# A digital twin for ^64^Cu production with cyclotron and solid target system

**DOI:** 10.1038/s41598-022-23048-5

**Published:** 2022-11-12

**Authors:** Lorenzo Isolan, Mario Malinconico, William Tieu, Courtney Hollis, Marco Testa, Matteo Melandri, Alessandro Brunetti, Marco Sumini

**Affiliations:** 1 Montecuccolino Laboratory, Industrial Engineering Department, University of Bologna, Via Dei Colli, 16, 40136 Bologna (BO), Italy; 2 National Institute for Nuclear Physics (INFN), Viale C. Berti Pichat, 6/2, 40127 Bologna (BO), Italy; 3 COMECER, Via Maestri del Lavoro, 90, 48014 Castel Bolognese, RA Italy; 4 The University of Adelaide, Adelaide, SA 5005 Australia; 5 Molecular Imaging & Therapy Research Unit (MITRU), South Australian Health and Medical Research Institute (SAHMRI), North Terrace, Adelaide, SA 5000 Australia

**Keywords:** Characterization and analytical techniques, Design, synthesis and processing

## Abstract

One method for finding reliable and cost-effective solutions for designing radioisotope production systems is represented by the “digital twin” philosophy of design. Looking at cyclotron solid targets, uncertainties of the particle beam, material composition and geometry play a crucial role in determining the results. The difference between what has been designed and what can be effectively manufactured, where processes such as electroplating are poorly controllable and generate large non-uniformities in deposition, must also be considered. A digital twin, where the target geometry is 3D scanned from real models, can represent a good compromise for connecting “ideal” and “real” worlds. Looking at the ^64^Ni(p,n)^64^Cu reaction, different Unstructured-Mesh MCNP6 models have been built starting from the 3D solid target system designed and put into operation by COMECER. A characterization has been performed considering the designed ideal target and a 3D scan of a real manufactured target measured with a ZEISS contact probe. Libraries and physics models have been also tested due to limited cross-section data. Proton spectra in the target volume, 3D proton-neutron-photon flux maps, average energies, power to be dissipated, shut-down dose-rate, ^64^Cu yield compared with various sources of experimental data and beam axial shifting impact, have been estimated. A digital twin of the ^64^Ni(p,n)^64^Cu production device has been characterized, considering the real measured target geometry, paving the way for a fully integrated model suitable also for thermal, structural or fluid-dynamic analyses.

## Introduction

With the growing demand of radionuclides for medical applications and the increasingly required high-quality standards, it has become necessary to look for new and efficient production systems^1^. Several radionuclides of interest were historically produced in research reactors or were the by-product of nuclear fuel reprocessing activities^2^. With the increasing shortage of these facilities and the shutdown of several of them^3^ the current production technology relies on a more standard and safely manageable accelerator-target approach, which can also be more freely developed at a commercial level^4^. This new industrial and market related approach makes the tuning of production facilities even more strategic: reliability, safety, timing, product quality, and isotope related extraction issues are becoming mandatory aspects to be analyzed looking at the overall efficiency of the production chain, from the setup of the beamline to the target post-processing. So, this implies that the process must exit from a classical “trial-and-error” approach in favor of a more rigorous production standard definition. One of the methods under development for finding reliable and cost-effective solutions in several industrial contexts is represented by the “digital twin” philosophy of design — the virtual representation of a physical entity of an arbitrarily complex system. For the actual problem, the innovation stands on the analysis of the beam-target system model^5,6^. More specifically, looking at a cyclotron as a beam source (a quite standard one) coupled with a solid target^1,7,8^, the design involves a quite complex configuration space, such as the source particle beam spectrum characterization, the material composition and characteristics of the target hosting device (the “shuttle”) and details like target tilting, shape, and thickness, degrader foil composition, all playing a crucial role regarding the production efficiency of the whole chain. This approach, which is becoming more effective due to the availability of efficient computational tools and HPC resources, is quite promising because it opens the possibility of the setup of a holistic optimization process in terms of both geometry and materials and creates a robust approach for experimental production data analysis. Finally, an interesting output is the possibility of a more critical interpretation of the available data on the particle beam cross-sections with the various elements and isotopes^9^ that often rely on poorly populated experimental data sets.

**Figure 1:**
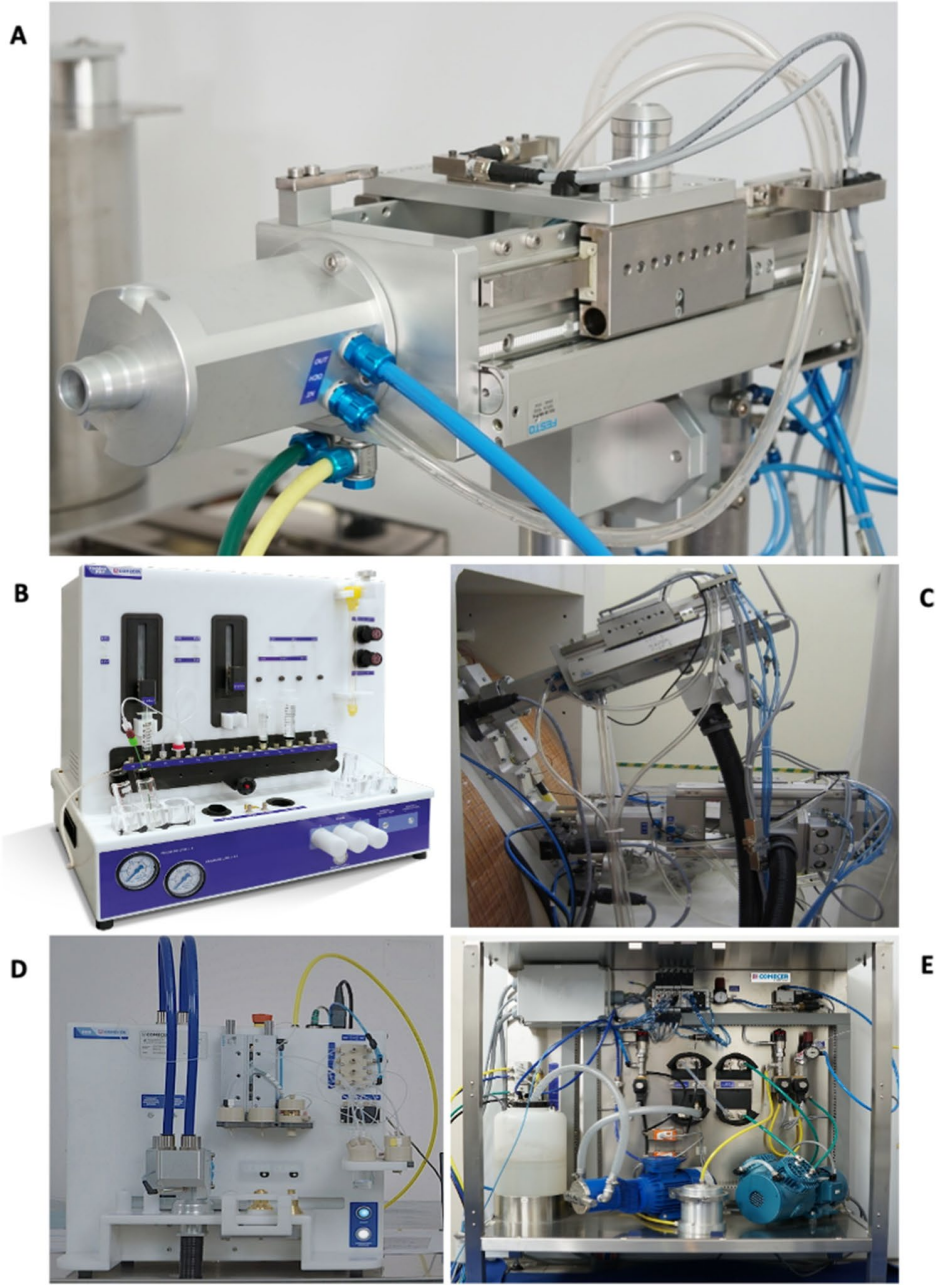
Panel (**A**), irradiation unit (PTS). Panel (**B**), TADDEO-PRF. Panel (**C**), coupling with two different PTS and a reference cyclotron. Panel (**D**), EDS. Panel (**E**), cooling system.

As reference case-study a very peculiar and well conceptualized system, ALCEO^10,11^, has been considered. Indeed, starting from 2008, COMECER has effectively prototyped and built an automated system implementing the ALCEO concept, which is based on the coupling between a beam extracted from a medical cyclotron and a suitably designed irradiation unit hosting a shuttle supporting a solid target. In a quite common implementation, the solid target is enriched ^64^Ni and the irradiation is optimized for the production of the ^64^Cu isotope through the nuclear reaction ^64^Ni(p,n)^64^Cu (other nuclear reactions using different target isotopes are also manageable using this system)^12–14^. See Fig. 1 for the actual configuration. The irradiation/production phase in ALCEO is complemented by an autonomous system for shuttle handling (Pneumatic Target System, PTS), target post-processing and radioisotope purification. After target irradiation, the target is automatically transferred to the Electrochemical Dissolution and transfer Station (EDS); here, together with the chemistry module (Taddeo Purification Radio-Pharmaceutical, PRF), the dissolution and purification process of the target takes place. At the end of the purification process, the desired radioisotope will be delivered to a synthesis module for labeling or directly to a shielded container.

This choice gives the possibility of an effective benchmark for the proposed “digital twin” here presented and discussed, focused on the analysis of the transport, collision and transmutation processes that take place in the irradiation unit of the beam-target system, the core component of the ALCEO device. This happens through the setup of a detailed model for the Monte Carlo^15^ transport code MCNP6^16,17^, based on the possibility of an Unstructured Mesh (UM) geometry domain^18^, that can be directly obtained from operative 3D CAD drawings or from real component scanning, also taking into account the timing of the irradiation phase. Moreover, MCNP6 benefits from the possibility of directly using several nuclear interaction models as event generators^19,20^. This helps in bypassing the nuclear data library build-up phase^21,22^, acting as a useful shortcut in those cases, like the one related to the actual problem, that show a quite dispersed cloud of experimental data for the involved isotopes, making cross-section evaluation difficult^23^. In this way the opportunity for a global and unifying assessment of a good number of already available theoretical and experimental results regarding ^64^Cu production via the ^64^Ni(p,n)^64^Cu reaction has been set, allowing the definition of a working phase space for the tuning of the device and of the production parameters and their engineering implementation. Moreover, the results here obtained can be easily extended to a whole class of radioisotope production chains based on the same beam-target technological approach^11,24,25^.

## Materials and methods

### The irradiation unit of the ALCEO system

The complete ALCEO system is shown in^10,11^. The irradiation unit coupling with the beam channel coming from a standard cyclotron (e.g., of medical application class Z11029002 or for R&D use) is shown in Supplementary Figure S1. The typical parameters of the cyclotron and of the beam are reported in Supplementary Table S1. The most relevant cyclotrons, already working together with the ALCEO solid target system, are the GE PETtrace (16.5 MeV), the IBA CYCLONE (from 15 to 30 MeV) or KIUBE cyclotron (18 MeV) and ACSI TR-19 (from 14 to 19 MeV) or TR-24 (from 18 to 24 MeV). The physical domain taken into consideration starts from the interface section that receives the proton beam channel in void conditions and ends with the components that implement the coupling with the water-cooling system. The whole unit has been transformed into its digital counterpart (see Supplementary Figure S2). Included into the model are all the constructive details like the gaskets, the void channel (the inner volume of the irradiation unit traveled by the beam up to the so-called degrader foil, see Supplementary Figure S1), the filling gases as the helium in front of the target, and the water used as coolant. The proton beam energy has been set to 16.5 MeV (the standard for GE PETtrace), as in operational conditions. To avoid long mean free paths and set the energy range of the beam equal to the one where cross-sections are more effective for the chosen nuclear reaction (see Supplementary Figure S3) the proton energy source is reduced thanks to an aluminum degrader foil installed in the PTS. In this way a good trade-off has been obtained with respect to thickness and mass of the target, making easier the post processing and extraction phase. For a cyclotron proton beam energy of 16.5 MeV, to degrade the beam to 14.5 MeV (the reference beam energy chosen in the ALCEO system design for the nuclear reaction ^64^Ni(p,n)^64^Cu), the degrader foil inserted in the PTS has a thickness of 320 µm. For cyclotrons with energies different from 16.5 MeV, the degrader foil thickness to degrade the beam energy to the selected one can be easily adjusted.

### The proton beam target

For the target material and geometry, two cases have been considered: an ideal configuration with a homogeneous nickel plating,

1. 7 mm plating diameter,
2. 50 mg plating mass,
3. 0.144 mm plating thickness,
4. 8.9 g cm^−3^ density,

and a real one, characterized by the uneven nickel distribution produced by an effective electroplating procedure.

**Figure 2:**
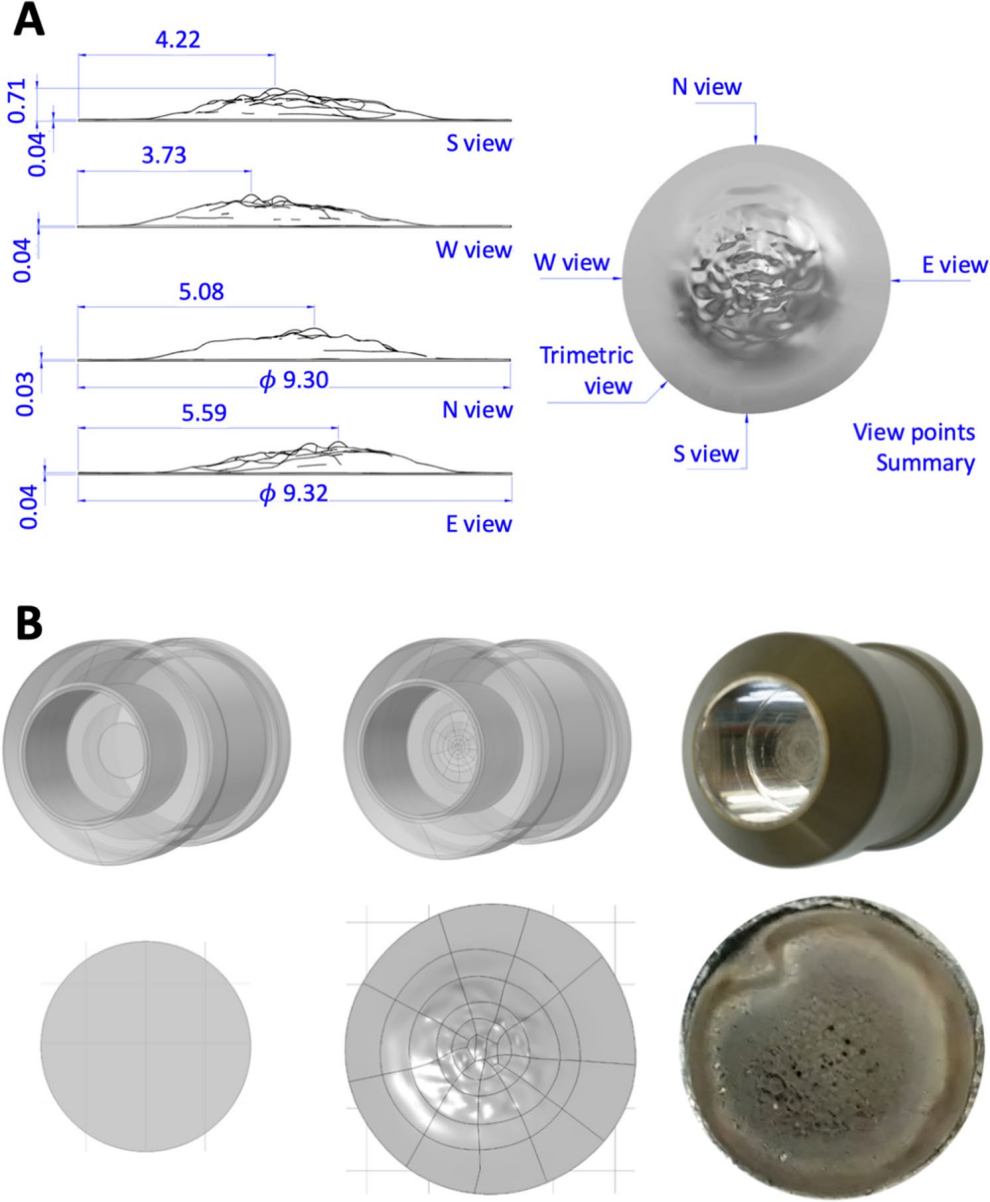
Panel (**A**), real target scanning results. Panel (**B**), Above, shuttle prospective view. Below, target’s planar view. Left, ideal model. Center, 3d scan of the effective target, showing also the irregular pattern, as transferred to the Monte Carlo Model (with some computational limits on the discretization of the geometric domain). Right, real target.

Due to the fact that Ni is electroplated on a Pt support and that Pt is a radiopaque material, a CT scan approach turns out to be not possible. So, the real target has been transformed into its digital twin through a scan system based on a high-precision Coordinate Measuring Machines (CMMs) contact tactile probe (ZEISS)^26,27^ that have the essential qualities required for high precision complete 3D capabilities with well-established accuracy. The result is the spatial domain shown in Fig. 2.

For the dimensional survey of the components under examination, the CMM instrument Hexagon TIGO SF—Sensor LSPX-1C with a continuous contact acquisition (scan acquired with a ruby microbead) has been used.

The acquisition parameters used were as follows: density 100 points/mm; speed and acceleration 0.1 mm/s; 120 radial scans at 3° angular offset. The SW supplied with the instrument makes possible to acquire the spatial coordinates, which were then processed with GEOMAGIC DESIGN X for the geometric reconstruction (and partial smoothing) in a STEP format.

1. 9.32 mm plating diameter,
2. 51.92 mg plating mass,
3. up to 0.750 mm plating thickness,
4. 5.62 g cm^−3^ density (estimated from the electroplated mass and the surface scan analysis).

See Fig. 2 for a visual comparison of the ideal target model and the real target. The scan volume shown in Fig. 2 panel A is transformed in the UM domain as in Fig. 2 panel B, undergoing one more unavoidable smoothing step. What becomes immediately apparent is the peaking and the anisotropy of the nickel distribution and the difference in density between the two models due to the unavoidable porosity of the real target: this has as an obvious consequence that the proton from the beam sees a quite different optical thickness^28^ and that translates to a different efficiency in terms of the transmutation rate.

### MCNP6 unstructured mesh model setup

Starting from the digital PTS unit’s model (Supplementary Figure S2) and from the 3D scan results (Fig. 2), the MCNP models have been set up with two target configurations: the ideal and the real geometries.

**Figure 3:**
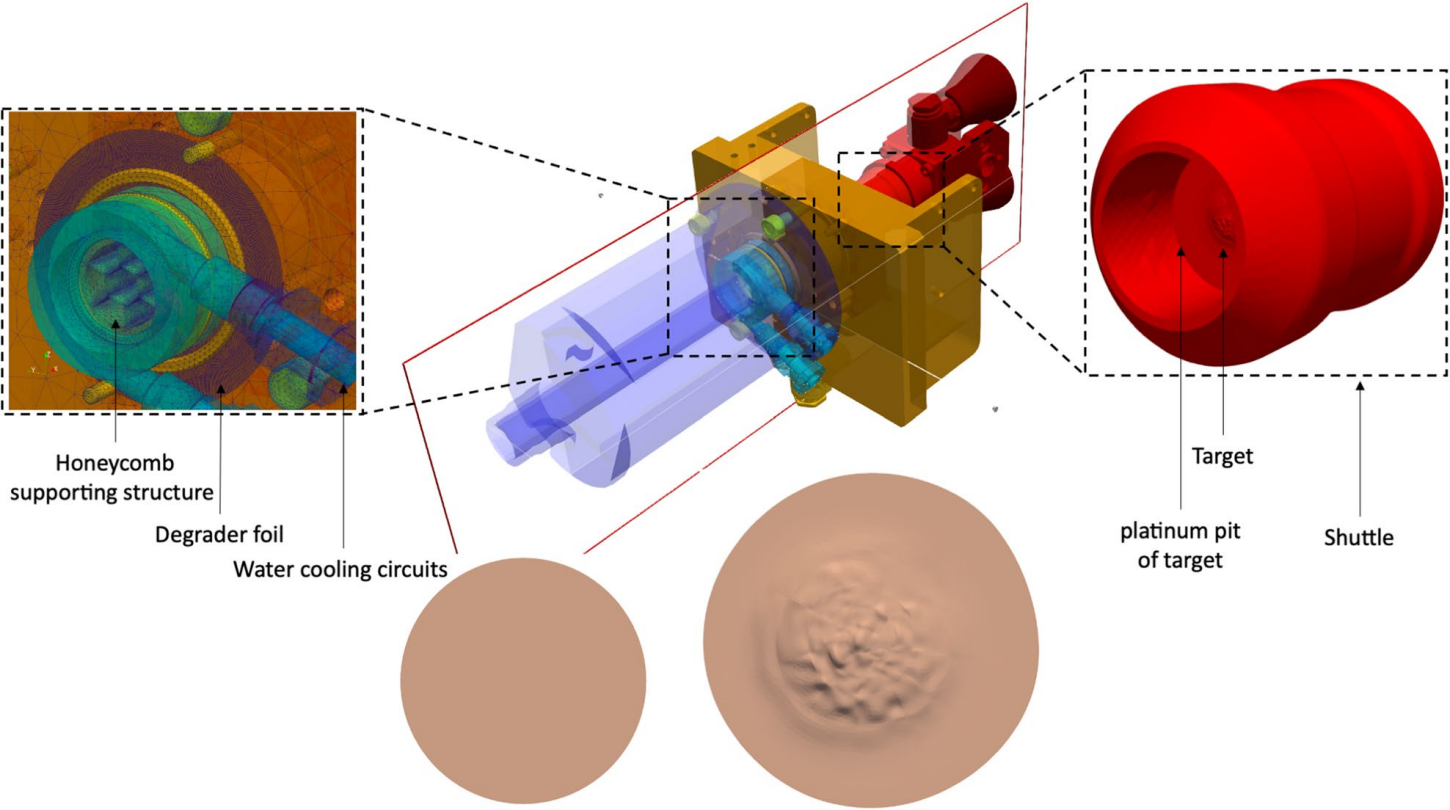
MCNP geometry details. Above, center: MCNP model of the whole PTS unit. Above, left: magnification of the coupling parts between the beamline extraction channel and the PTS (water cooling circuits and the so-called honeycomb supporting structure are visible together with the degrader foil). Above, right, shuttle hosting the target. Below, from left to right: ideal and real target as modeled in MCNP. Cut plane at the proton beamline and target axis is shown.

To avoid unnecessary bias and sources of uncertainty, the proton beam has been modeled as a 16.5 MeV uniform source of circular section with a 3.7 mm radius and 1 h of constant irradiation. In both cases, the whole irradiation unit has been transformed in an Unstructured Mesh (UM) domain using the COMSOL meshing tool^29^ (see Supplementary Figure S4), then moved as a set of assemblies in an ABAQUS input style that can be managed (as an ASCII file) to become ready, after the proper assignment of materials (Supplementary Table S2^30^), to be submitted to the *um-pre-op* tool for generating the Monte Carlo input file to be run with the MCNP6 code (see^18^ for details), see Fig. 3. In Supplementary Table S3 the typical simulation performances for the two models are shown, demonstrating the good agreement between the two discretized domains.

### MCNP6 nuclear data setup

As can be seen from Supplementary Figure S3 (from references^31–33^), the experimental data for ^64^Cu production from the ^64^Ni(p,n)^64^Cu reaction are quite dispersed, with some relevant uncertainties, and not very well fitted by the cross section available in the libraries. A significant point is the extremely poor performance in the energy range of interest of the ENDF libraries (VI, VII and VIII), that still mainly rely on outdated models. Better performance can be obtained by the TALYS models (but going through the buildup of the TENDL libraries)^32,34^. So, it appeared more efficient to directly use all the available models as the event generator as allowed by the MCNP6 code. The various options range from the CEM03.03^35^ to the ISABEL^36^, BERTINI^37^ and INCL4^38^ models (see also^17^).

### MCNP6 tallying process setup

Different tallies were specified in Monte Carlo calculations as different results have to be scored:

1. *Proton spectrum in the target volume*: the proton spectrum in the target volume has been estimated with an F4 tally in combination with the E card for an energy binning from 0.01 (cutoff energy as implemented thanks to the PHYS card) to 16.5 MeV, discretizing the domain in 100 parts (also, for the spectrum estimation, cross sections and models have been checked and controlled by the PHYS card: TENDL-2019 library, and CEM03.03, ISABEL, BERTINI and INCL4 models);
2. *Proton average energy in the target volume*: the results from the F4 tally were also used for evaluating the proton mean energy (in the target volume) through the sum of the energies weighted by the related probabilities;
3. *Power to be dissipated in the target volume*: the power to be dissipated from all particles (primary beam and secondary particles produced through the interaction processes) has been estimated with an F8 tally;
4. *3D fluxes and radionuclides yield estimate*: for the 3D quantities evaluations in the UM domains, the special EMBEE tally card has been used with the 4 option (flux estimator) for protons, secondary neutrons and photons, embedding the model universes thanks to the EMBED keyword (post-processing the results with MATLAB and PARAVIEW^39,40^); for protons, the shifting of the particle beam axis from 0 to 3 mm for simulating the oscillating behavior as experienced during experiments (and performing a sensitivity analysis) has also been considered for evaluating the ^64^Cu yield with the CEM03.03 physics model. The estimate in terms of ^64^Cu production (as mCi μA^−1^ h^−1^) was then also performed both by using the FT8 RES special treatment tally with MCNP physics models (CEM03.03, ISABEL, BERTINI, INCL4) and also with the Reaction Rate channels from cross section library TENDL 2019;
5. *Shut-down Dose-Rate*: The shut-down dose-rate has been evaluated with a collision heating tally in combination with the Tally-Time card T (in "shakes" as measurement’s unit, equal to 1.0E-08 s), starting from the beginning of the irradiation (lasting 1 h) up to 72 h and including delayed particles. The Activation Control (ACT) combined with an F8 tally and the special treatment RES keyword in an FT8 card has also been considered as a strategy for obtaining the isotope list generated by bombarding the target.

See^15,17,18^, for further tallying process details.

Moreover, as a choice, any bias about collision and transmutation processes has been avoided, trying to obtain a proper convergence in an ideal simulation condition. As it is well known, Monte Carlo modeling is efficient in tracking particle collisions, but rare events can hardly be managed. To avoid issues in particle tracking different choices are available for the users: increasing the number of simulated histories can be the best option for maintaining coherence with the simulated physics as no forced collisions are introduced^15^. In the present work, the direction that has been followed has been to increase the number of simulated particles as the model converged in a reasonable time under the Relative Error (RE) as suggested by the code manual (see^16^ for details).

## Results

**Figure 4:**
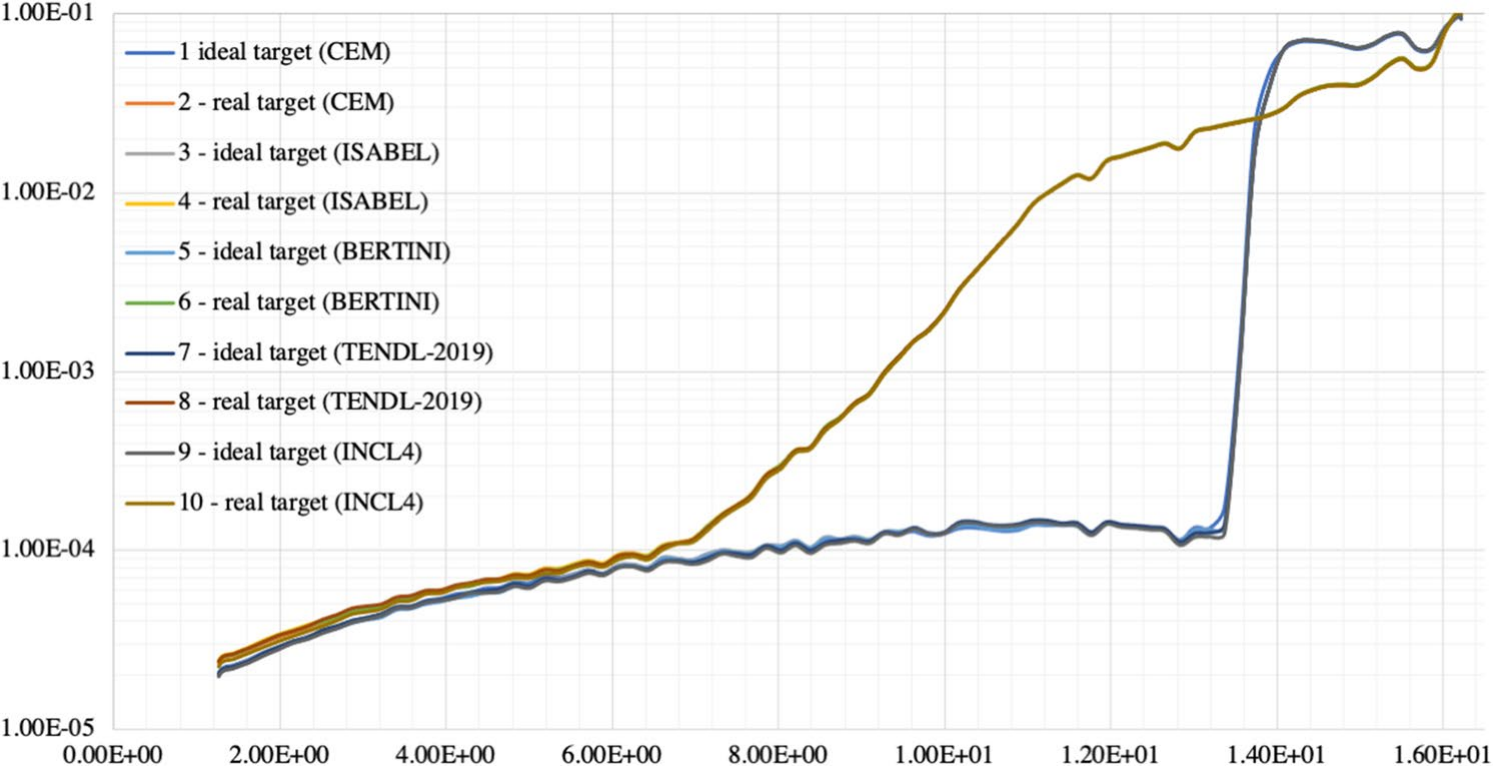
Proton spectra in the target volume. Y axis, intensity [a.u.], X axis, energy [MeV]. As it can be seen, from the ideal and the real target, the tallied spectra remain coherent between models and cross sections.

**Figure 5:**
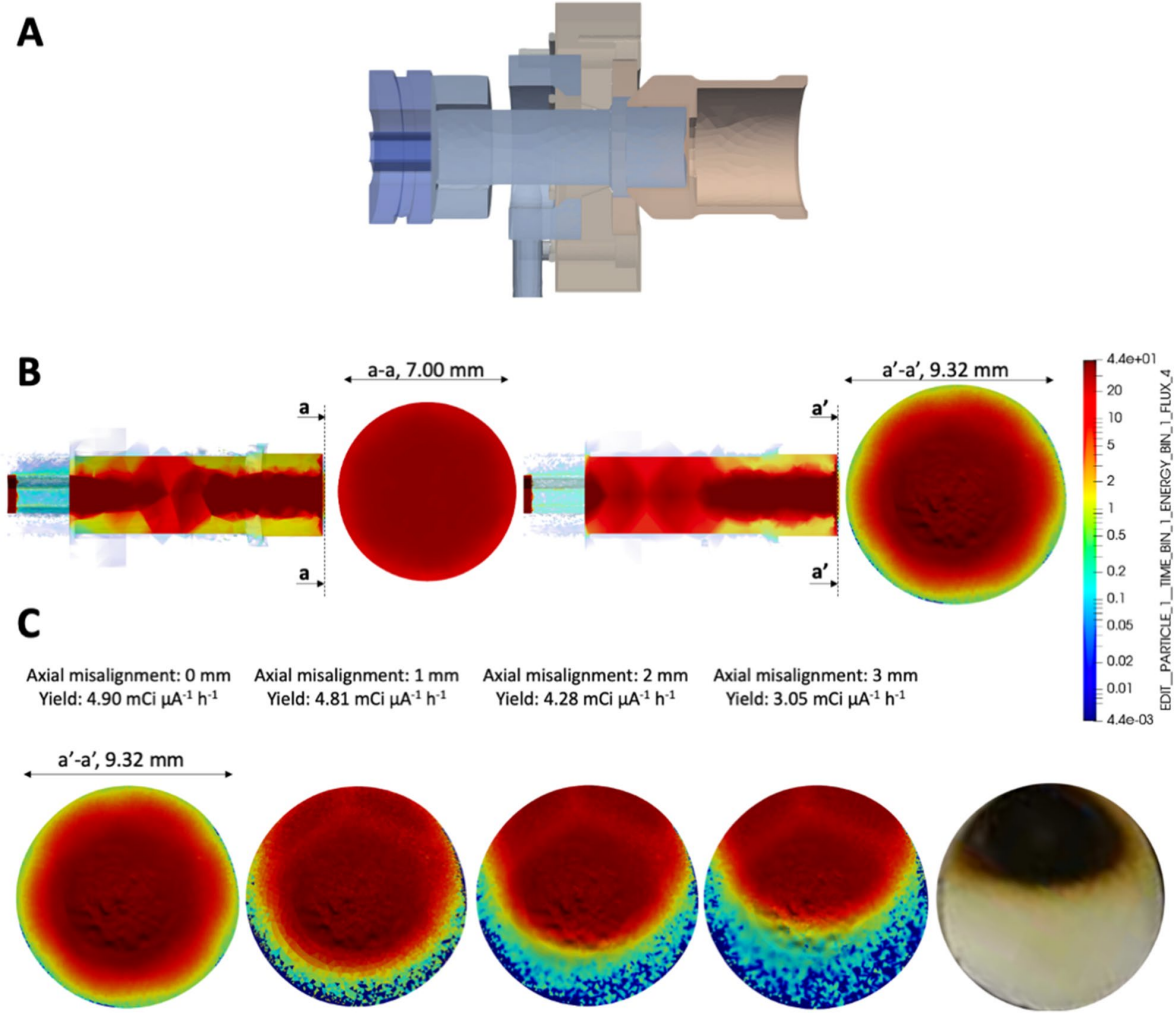
Panel (**A**), cut view along the proton beamline (See Fig. 2 for details of the 3D model and the cut plane). Panel (**B**), proton flux, arbitrary units. CEM03.03 model only taken as reference. Left, ideal target; Right, real target. Planar and PTS relevant section views are displayed. Panel (**C**), axial misalignment sensitivity analysis and comparison with a typical COMECER paper burn experimental result. The honeycomb supporting structure shadow is clearly visible (see also Supplementary Figure S1 and Fig. 3 for details).

**Figure 6:**
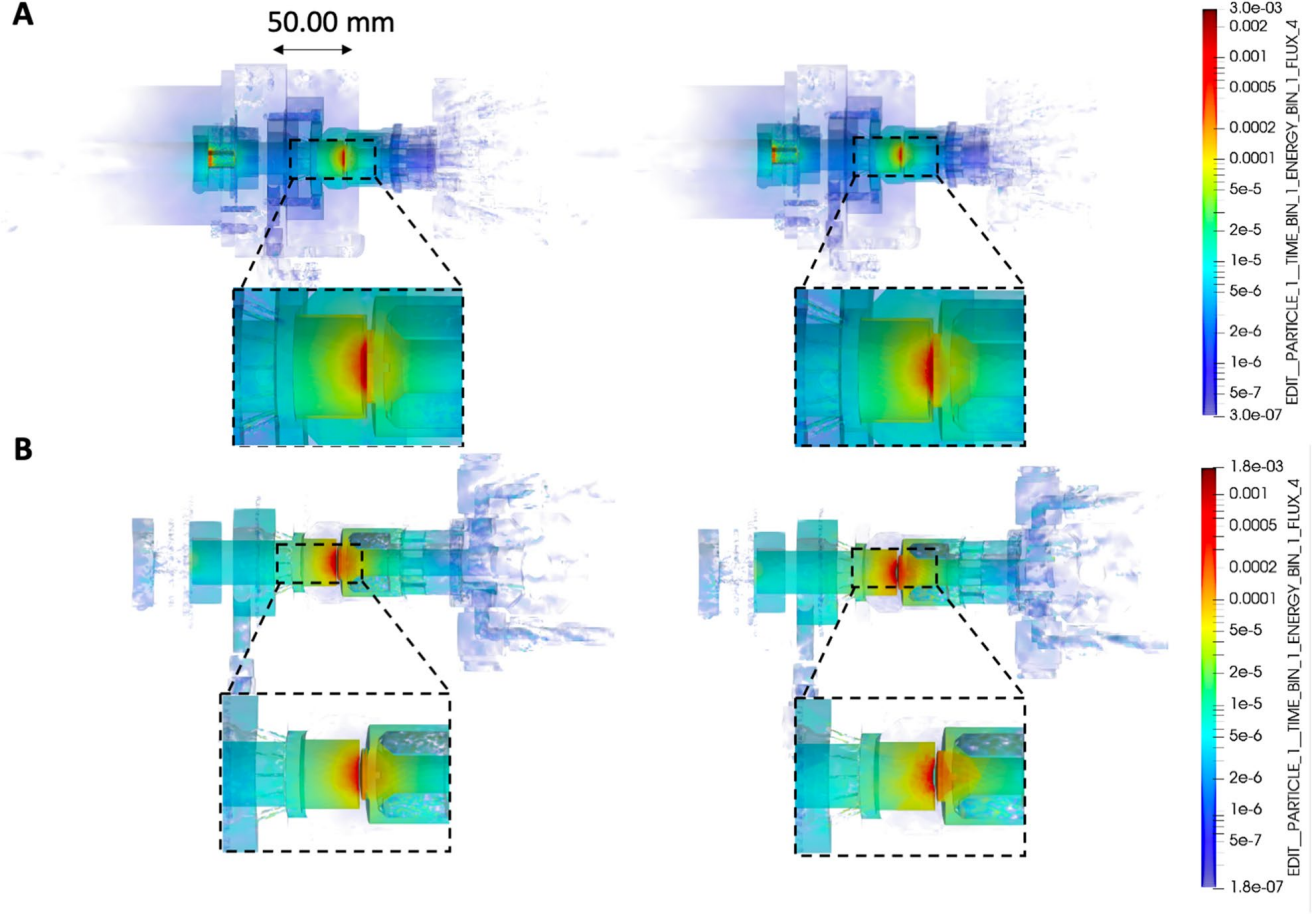
Panel (**A**), photon flux, arbitrary units. CEM03.03 model only taken as reference. Left, ideal target; Right, real target. Above, PTS relevant parts section; Below, zoom around the target region. Panel (**B**), neutron flux, arbitrary units. CEM03.03 model only taken as reference. Left, ideal target; Right, real target. Above, PTS relevant parts section; Below, zoom around the target region.

**Figure 7:**
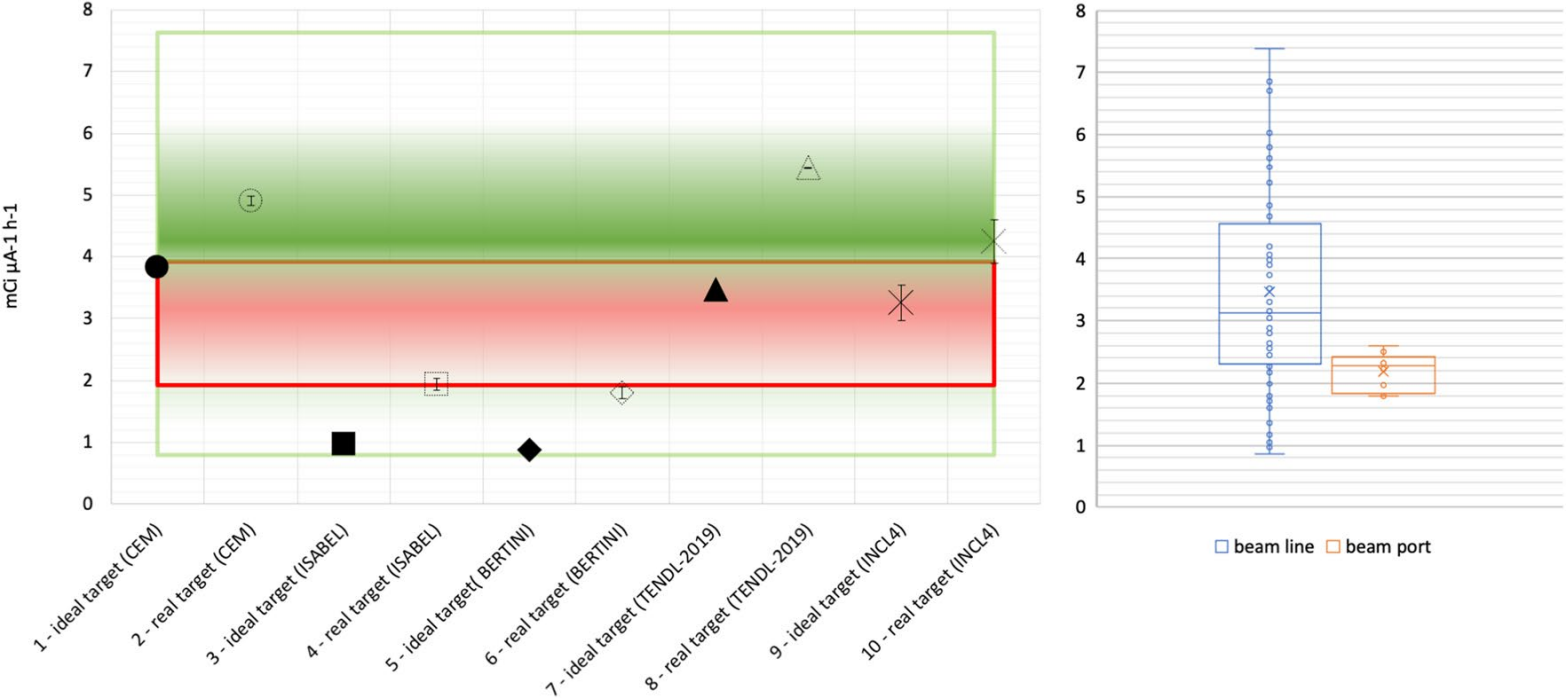
Left, ^64^Cu yield estimated with cross sections and model physics for ideal and real targets. Red band indicates the values experimentally found by COMECER with the ALCEO system mounted on cyclotron beam ports (the higher the color intensity, the higher the frequency of the experimentally found activity; color band centered on the 2.5 value). The green band instead is referred to a PTS device directly interfaced with the beam line. Right, experimental data obtained by COMECER. Chart showing results for beam line (blue) and beam port (orange) together with experimental values (dots), mean (cross), local minimum and maximum (horizontal rays) and the rectangle showing median and 25th-75th percentiles as in the usual “Box and Whisker” representation. Y axis, mCi μA^−1^ h^−1^.

**Figure 8:**
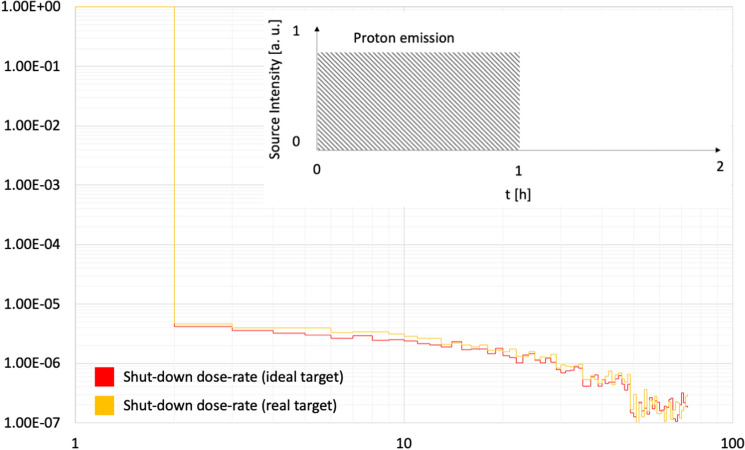
Normalized shut-down dose-rate from the end of beam up to 72 h. Red, ideal target. Yellow, real target. Y-scale in arbitrary units. X axis, time [h].

1. *Proton spectrum in the target volume*
  The proton spectrum tallied in the target volume, demonstrates the coherence between models and cross sections and is shown in Fig. 4.
2. *Proton average energy in the target volume*
  The average proton energy estimated in the target volume starting from the CEM3.03^26^ spectrum is shown has been found to be 15.1 MeV (Relative Error, R.E., 0.0003^16,17^) and 14.4 MeV (R.E., 0.0002) for the ideal and the real cases respectively.
3. *Power to be dissipated in the target volume*
  The power to be dissipated from the target volume (from all particles, e.g., n, p, h, e, a, # and with the CEM3.03 model only taken as reference) corresponds to 1.93 W μA^−1^ (R.E. 0.0002) and 2.12 W μA^−1^ (R.E., 0.0004) for the ideal and real cases respectively.
4. *3D fluxes and radionuclides yield estimate*
  For the two studied cases, 3D proton fluxes have been obtained, see Fig. 5. Moreover, trying to recreate real conditions, a beam axial shifting from 0 to 3 mm has been considered, observing that the ^64^Cu yield evaluated with the CEM03.03 physics model for the real target decreases up to 40%, see Fig. 5. A further benchmark has been performed starting from COMECER data extracted from the^10^ work, where a 3 mm misalignment implies a reduced yield of 3.36 mCi μA^−1^ h^−1^ in good agreement with the value obtained with MCNP (3.05 mCi μA^−1^ h^−1^). Figure 5 also shows a *paper burn* (a simple but effective check procedure) obtained by COMECER in similar misalignment conditions. The run performed with the CEM03.03 with the ACT card also assess the probability for production of ^61^Fe, ^61^Co, ^63^Ni, ^65^Ni, ^62^Cu as isotopes generated in the target volume from the ancillary nuclear reactions ^64^Ni(n,a)^61^Fe, ^64^Ni(p,a)^61^Co, ^64^Ni(n,2n)^63^Ni, ^64^Ni(g,n)^63^Ni, ^64^Ni(n,g)^65^Ni, ^62^Ni(p,n)^62^Cu. These isotopes work as contaminants/impurities with respect of the extraction chemical procedure.
  3D photon-neutron fluxes have been evaluated as shown in Fig. 6.
  The ^64^Cu yields estimated using TENDL-2019 cross-section library, CEM03.03, ISABEL, BERTINI and INCL4 physics models for the ideal target were 3.4, 3.8, 0.9, 0.8, 3.2 mCi μA^−1^ h^−1^ and for the real target were 5.4, 4.9, 1.9, 1.8, 4.2 mCi μA^−1^ h^−1^ respectively, see Fig. 7. Experiments in beam line and beam port have also been considered for comparison.
5. *Shut-down Dose-Rate*
  The shut-down dose-rate due to the decay chains tallied up to 72 h after the End Of Beam (EOB) were slightly higher with the real target than the ideal target (16% average increase, see Fig. 8). Both photon and neutron fluences (Fig. 6) and the output of the decay processes (Fig. 8) could become of paramount importance when dealing with unwanted activation events and radiation protection issues.

## Discussion

Some experimental and theoretical yield data are available for different primary beam energies and target setups as a result of target irradiations or cross-section analysis. In reference^7^, a 54 mg ^64^Ni target was irradiated with a 15.5 MeV proton beam and a yield of 5 mCi μA^−1^ h^−1^ was measured while 10.5 mCi μA^−1^ h^−1^ was predicted (a ratio of 2.1 between measured and predicted yield). Szelecsényi and Blessing^12^ irradiated a ^64^Ni target with energies between 9 and 12 MeV measuring a ^64^Cu yield of 6.4 mCi μA^−1^ h^−1^ and in the same work showed the prediction on a thick target with a 14.4 MeV beam equal to 13 MeV. Obata et al.^8^ bombarded a 48 mg ^64^Ni target with 12 MeV protons, obtaining a 6.5 mCi μA^−1^ h^−1^ yield against the 10.8 mCi μA^−1^ h^−1^ that was predicted (a ratio of 1.66 between the two values). Aslam et al.^13^ predicted 13 mCi μA^−1^ h^−1^ with a 14.4 MeV beam on a thick target while^23^ predicted 8 mCi μA^−1^ h^−1^. In reference^4^, an energy variable between 9 and 14 MeV was used and a ^64^Cu buildup was found to be 6.5 mCi μA^−1^ h^−1^. Poignant et al.^41^ approached the problem from the Monte Carlo perspective and reproduced the irradiation conditions of^7,8,12^, reporting ratios between the simulated yields and the experimental yields between 1.2 and 2.7, according to the theoretical values and overestimating the facilities results. As observed, the outcomes are unexpected, and the results are controversial due to a large divergence between theoretical estimation and experimental reality. At this stage, it is legitimate to attribute such differences to beam axial misalignment or shifting as well as mechanical defects in processes such as electroplating (for instance with respect to target density). Indeed, it has been demonstrated in the present work that the expected density between the ideal target and the real ^64^Ni deposit decreased from 8.90 to 5.62 g cm^−3^ and, as shown by the paper burn technique, axial shifting up to 3 mm can be expected (reducing the simulated yield from 4.90 to 3.05 mCi μA^−1^ h^−1^ with a reduction in productivity of almost 40%, the latter value is in accordance with typical experimental data from COMECER^10^).

Experiments performed with the PTS installed directly to a cyclotron beam port or connected to a beam line (in both cases using a GE PETtrace) (Fig. 7) have also been considered. The beam line, with its additional magnets, stabilizes the beam during the proton bombardment, avoiding, in part, the beam shifting effect. Moreover, because the beam line is far away from the other targets (gas, liquid) installed to the beam ports of the cyclotron, it’s less sensitive to any vibration (caused for example during the maintenance of the targets) which could increase, over time, the axial misalignment of the irradiation unit (PTS) to the beam of the cyclotron. Where the PTS was installed directly to the cyclotron beam port, irradiating a 45.25 ± 5.27 mg ^64^Ni target with a 14.5 MeV proton beam gave a ^64^Cu yield of 2.19 ± 0.30 mCi μA^−1^ h^−1^ (N = 10). In comparison, where the PTS was installed on a beam line, irradiating a 65.11 ± 16.42 mg ^64^Ni target with a 14.5 MeV proton beam gave a ^64^Cu yield of 4.20 ± 1.33 mCi μA^−1^ h^−1^ (N = 48). The experimental data demonstrates a decrease in ^64^Cu yield of up to 50% if the beam shifting effect is present (i.e., if the PTS is mounted directly to the cyclotron beam port), which correlates with the digital twin model simulation results of a 40% decrease in yield.

The digital twin could also be useful for evaluating not only how the primary beam interacts with the irradiation unit but also could clearly display how the secondary particles have been produced in terms of 3D spatial distribution (Fig. 6) and to clarify the shut-down dose-rate of the device (Fig. 8), helping in solving criticalities with respect to the radiation protection issues.

## Conclusions

A digital twin of the ^64^Ni(p,n)^64^Cu irradiation unit as designed by COMECER for the ALCEO production system has been fully characterized taking into account real data as the target geometry, paving the way to a fully integrated model suitable for thermal, structural or fluid-dynamic analyses.

The extensive use of models as event generators in MCNP has proven quite effective and, in future, a tuning between models and cross section libraries through dedicated V&V tools (for instance adapting to protons the recently made available JADE tool^42,43^) could be of great utility.

As shown in Supplementary Figure S5, the digital twin just discussed perfectly works as a transfer function between the external source characteristics and the post irradiation processing. It makes possible to establish a direct relationship between kind of cyclotron beam characteristics and profiles, materials and, last but not least, the post-processing constrains. In this way, it become possible to establish some formal and rigorous correlation between the physical and technological parameters that define the possible yield of the whole process and the unavoidable economical constrains. Thinking of improving the radioactivity yield, a sensitivity analysis on the parameters space phase can be considered with the aim to optimize the whole irradiation process.

However, as a first guess, it is quite evident that a better control of the electroplating deposition and target density, together with the use of beams coming from more stable sources could help in improving the yield without a dramatical change of the production chain.

## Supplementary Information

Supplementary Information.

## Data Availability

The datasets generated during and/or analyzed during the current study are available from the corresponding author on reasonable request.
