# Supplementary material for: A digital twin for 64Cu production with cyclotron and solid target system
Source: Sci Rep. 2022 Nov 12;12:19379. doi: 10.1038/s41598-022-23048-5 (PMC9653460; doi:10.1038/s41598-022-23048-5)
Supplement: Supplementary file 1 — Supplementary Information. [file 41598_2022_23048_MOESM1_ESM.docx]

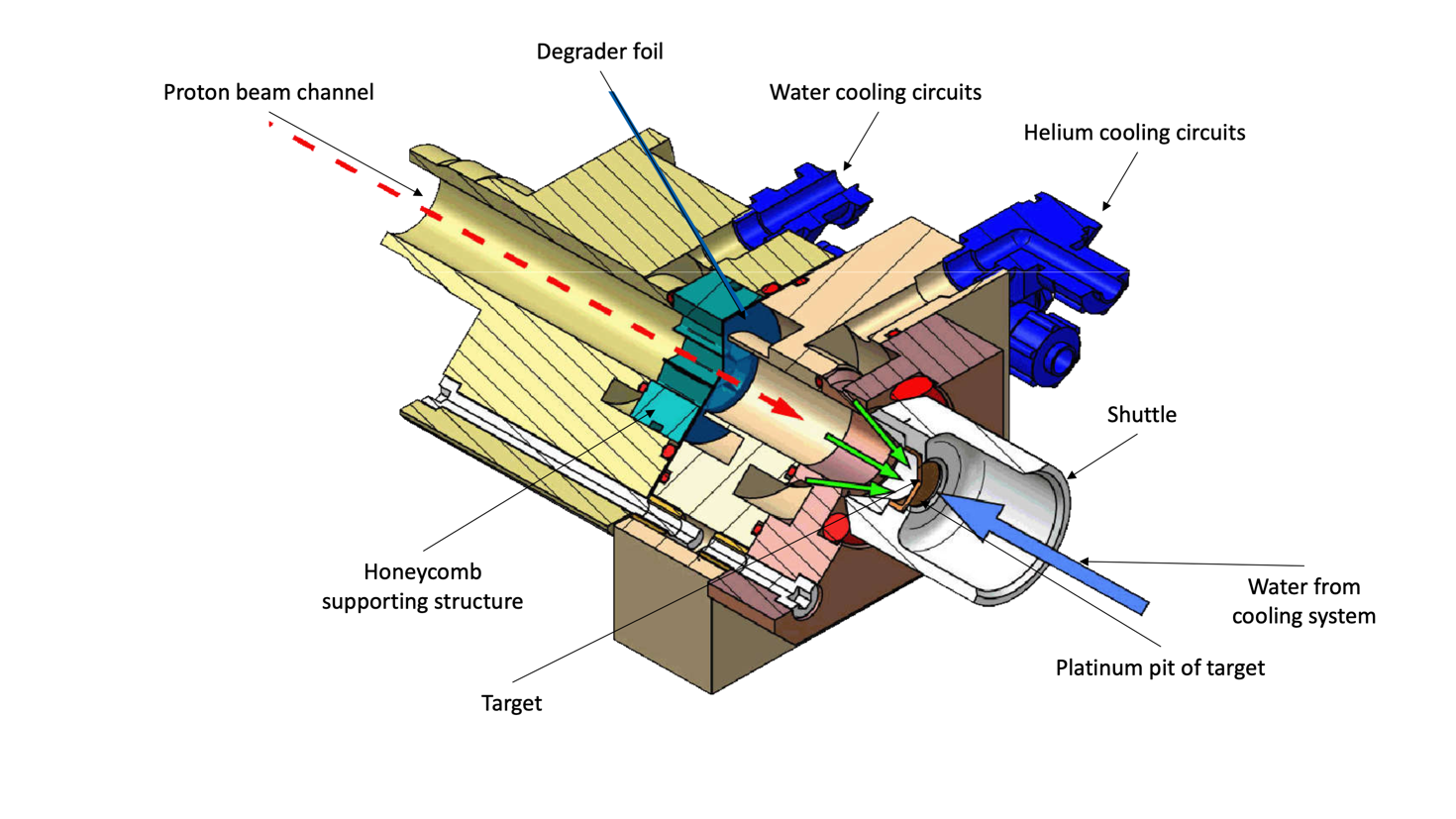


Supplementary Figure S1. Cutaway diagram of the PTS. Red arrow, proton beam; green arrows, helium flow in front of the target; strong blue arrow, water cooling behind the target’s platinum pit. Also, the gaskets are shown and considered in the MCNP model.


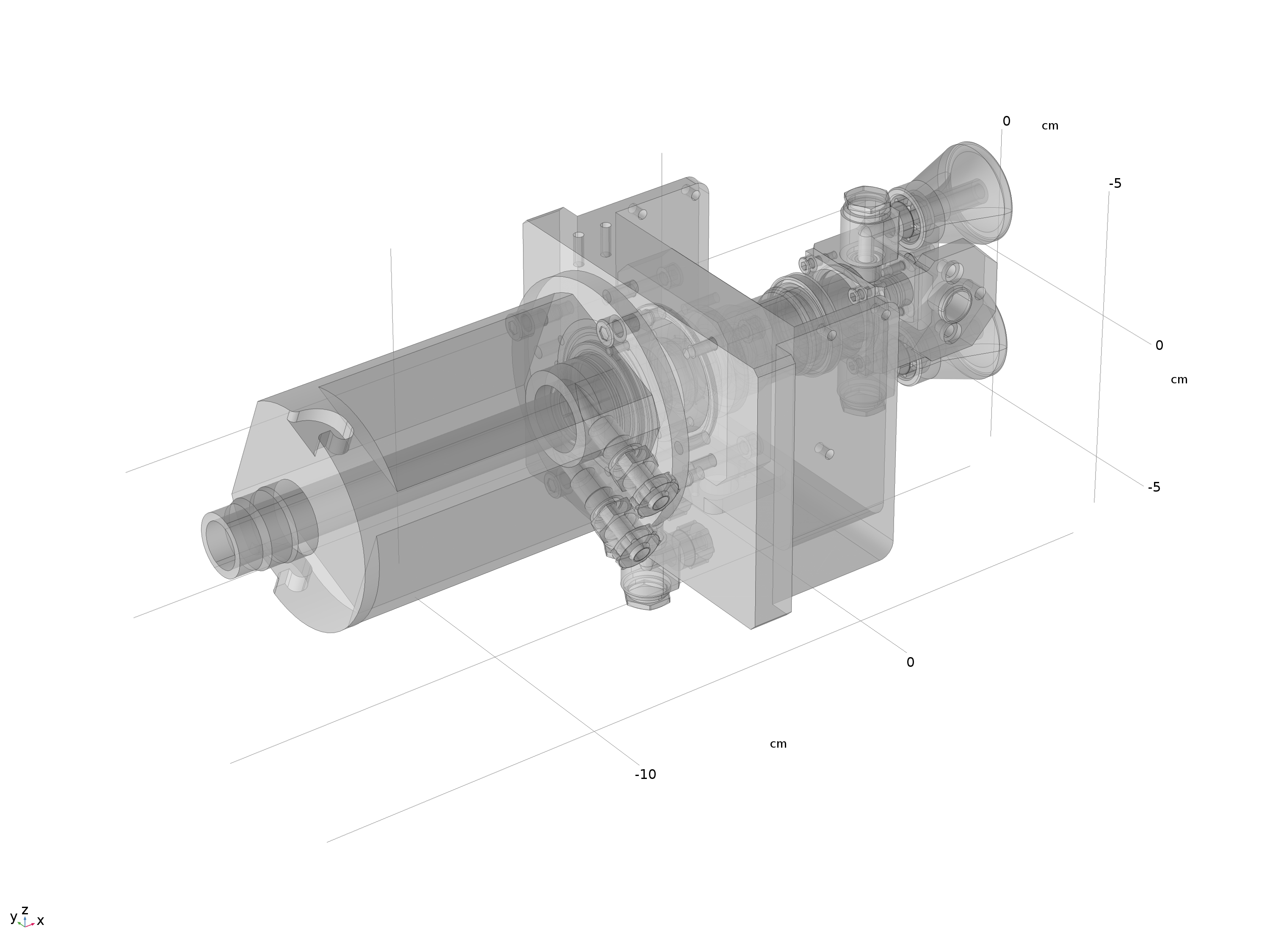


Supplementary Figure S2. PTS unit digital representation.


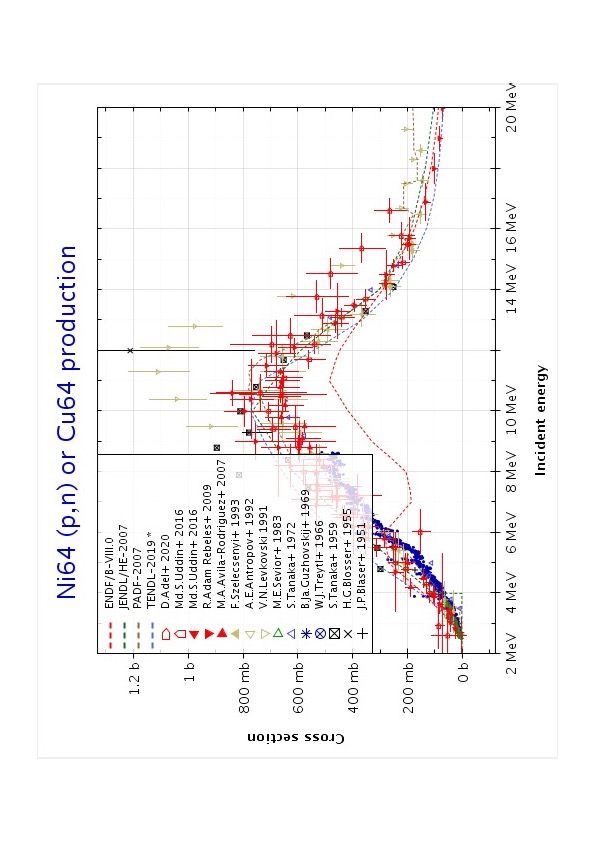


Supplementary Figure S3. MCNP F4 tally + Fm multiplier with MT reaction channel from XS. TENDL-2019 Based on TALYS interaction models, ENDF formatting [31][32]. ENDF/B-VIII.0 still relying on ENDF/B-VI MOD 1 release, mainly based on interaction models but with some limitations (quoting ENDF/B-VI MOD 1 Evaluation, Sept. 1997, S. Chiba et al.: “…The data for natural Ni were also used because there was not enough data for Ni-64 at MeV region interaction models”). Plot generated with the Janis tool [33].


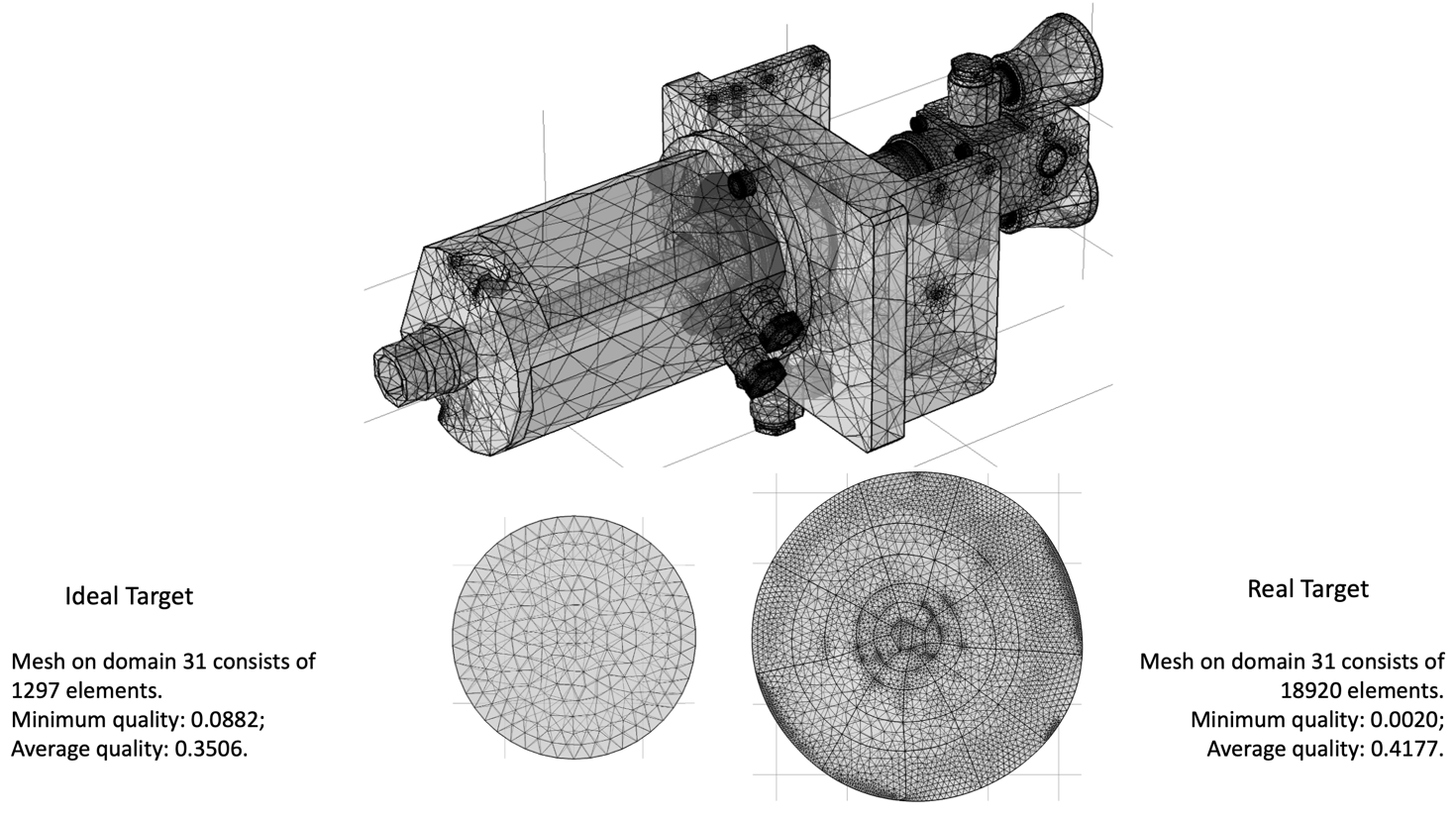


Supplementary Figure S4. Unstructured mesh domains.


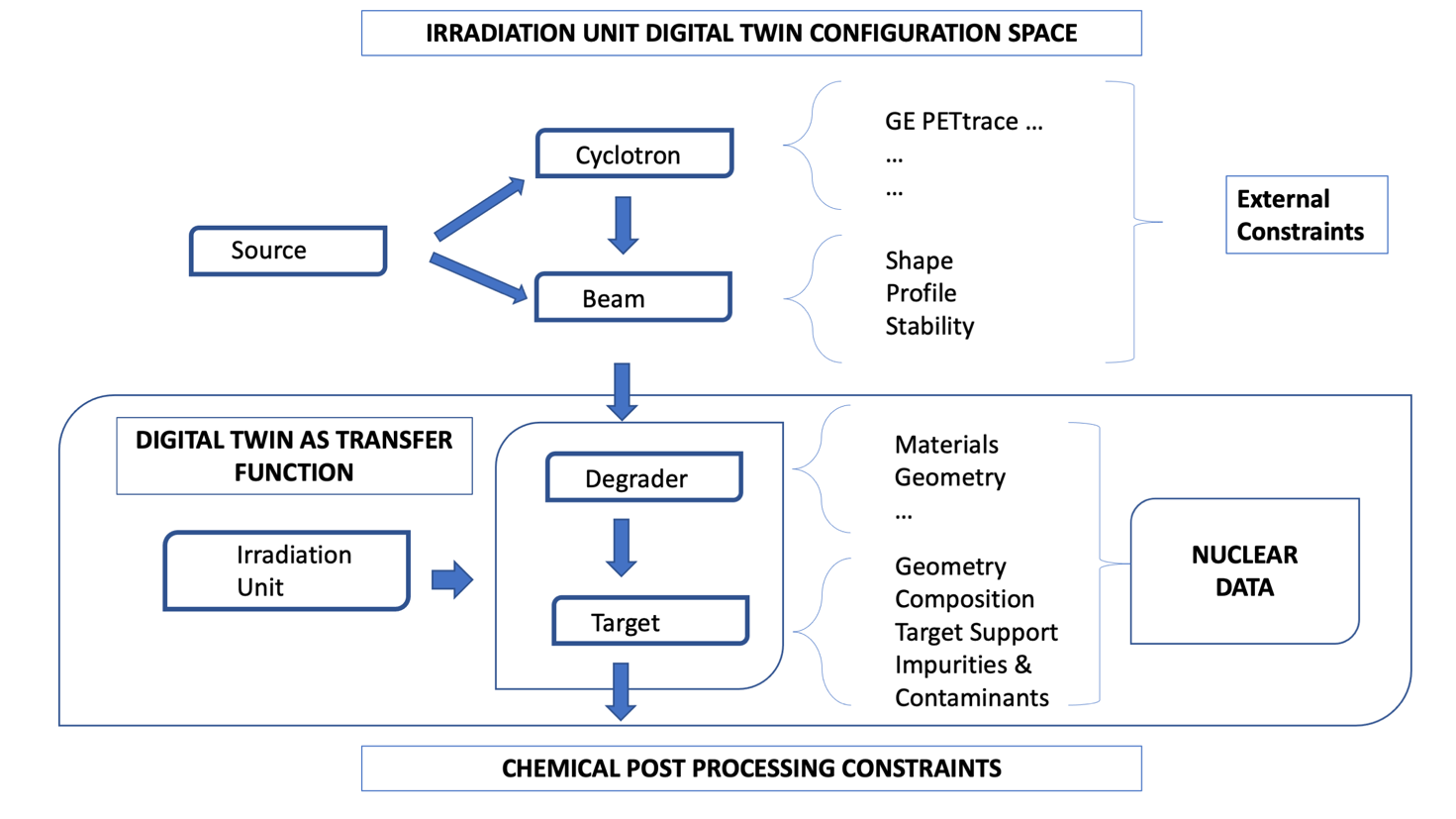


Figure 5. Irradiation unit digital twin configuration domain.

Supplementary Table S1. Typical cyclotron and proton beam parameters.

| GE PETTrace | IBA CYCLONE | IBA KIUBE | ACSI TR-19 | ACSI TR-24 |
| --- | --- | --- | --- | --- |
| 30-50 µA | 30-50 µA | 30-50 µA | 30-40 µA | 30-40 µA |
| 16.5 MeV | 15-30MeV | 18MeV | 14-19 MeV | 18-24 MeV |

Supplementary Table S2. Characteristics of the simulated MCNP model materials (weight fractions). Values truncated at the first decimal. ^(1)^atom fraction. ^(2)^Measured real target density (for the ideal target, the design value has been used: 8.9E+0 g cm^-3^). ^(3)^Viton^TM^ Fluorinated elastomer. ^(4)^if ZAID ends with “000”, the natural composition has been picked from the MCNP material library, otherwise the specific isotope is provided. See [30] for details.

| Element | Isotope  ZAID^(4)^ | Material | | | | | | | | | | | |
| --- | --- | --- | --- | --- | --- | --- | --- | --- | --- | --- | --- | --- | --- |
|  |  | Aluminum  Alloy | Pet | Water | Viton^TM,(3)^ | Stainless  Steel | Polycarbonate | Cooling  gas | Target  Support | Target^(1)^ | Silicon | Pvc | Beamline  (vacuum) |
|  |  | $\varrho$ [g cm^-3^] | | | | | | | | | | | |
|  |  | 2.7E+0 | 1.3E+0 | 1.0E+0 | 1.8E+0 | 8.0E+0 | 1.2E+0 | 3.0E-4 | 2.1E+1 | 5.6E+0^(2)^ | 2.3E+0 | 1.4E+0 | 1.2E-10 |
| H | 1001 |  | 4.2E-2 | 1.1E-01 | 9.4E-3 |  | 5.5E-2 |  |  |  |  | 4.8E-2 |  |
| He | 2004 |  |  |  |  |  |  | 1.0E+0 |  |  |  |  |  |
| C | 6000 |  | 6.2E-1 |  | 2.8E-1 | 4.0E-4 | 7.5E-1 |  |  |  |  | 3.8E-1 |  |
| N | 7014 |  |  |  |  |  |  |  |  |  |  |  | 7.5E-1 |
| O | 8016 |  | 3.3E-1 | 8.8E-01 |  |  | 1.8E-1 |  |  |  |  |  | 2.3E-1 |
| F | 9019 |  |  |  | 7.1E-1 |  |  |  |  |  |  |  |  |
| Mg | 12000 | 9.0E-2 |  |  |  |  |  |  |  |  |  |  |  |
| Al | 13027 | 7.4E-1 |  |  |  |  |  |  |  |  |  |  |  |
| Si | 14000 | 1.0E-2 |  |  |  | 5.0E-3 |  |  |  |  | 1.0E+0 |  |  |
| P | 15031 |  |  |  |  | 2.3E-4 |  |  |  |  |  |  |  |
| S | 16000 |  |  |  |  | 1.5E-4 |  |  |  |  |  |  |  |
| Cl | 17000 |  |  |  |  |  |  |  |  |  |  | 5.6E-1 |  |
| Ar | 18036 |  |  |  |  |  |  |  |  |  |  |  | 3.9E-5 |
|  | 18038 |  |  |  |  |  |  |  |  |  |  |  | 8.0E-6 |
|  | 18040 |  |  |  |  |  |  |  |  |  |  |  | 1.2E-2 |
| Ti | 22000 | 1.0E-2 |  |  |  |  |  |  |  |  |  |  |  |
| Cr | 24000 | 2.5E-2 |  |  |  | 1.9E-1 |  |  |  |  |  |  |  |
| Mn | 25000 | 4.0E-2 |  |  |  |  |  |  |  |  |  |  |  |
|  | 25055 |  |  |  |  | 1.0E-2 |  |  |  |  |  |  |  |
| Fe | 26000 | 5.0E-2 |  |  |  | 7.0E-1 |  |  |  |  |  |  |  |
| Ni | 28000 |  |  |  |  | 9.2E-2 |  |  |  |  |  |  |  |
|  | 28058 |  |  |  |  |  |  |  |  | 7.5E-2 |  |  |  |
|  | 28060 |  |  |  |  |  |  |  |  | 3.4E-2 |  |  |  |
|  | 28061 |  |  |  |  |  |  |  |  | 4.0E-3 |  |  |  |
|  | 28062 |  |  |  |  |  |  |  |  | 5.5E-1 |  |  |  |
|  | 28064 |  |  |  |  |  |  |  |  | 9.9E+1 |  |  |  |
| Cu | 29000 | 1.0E-2 |  |  |  |  |  |  |  |  |  |  |  |
| Zn | 30000 | 2.0E-2 |  |  |  |  |  |  |  |  |  |  |  |
| Pt | 78000 |  |  |  |  |  |  |  | 1.0E+0 |  |  |  |  |

Supplementary Table S3. Figure Of Merit (FOM) and convergence parameters (Variance Of Variances, VOV) typical comparison.

|  | NPS | Mean | Error | VOV | FOM |
| --- | --- | --- | --- | --- | --- |
| Ideal target | $\cong$1.0E+07 | 1.8459E-03 | 0.0073 | 0.0001 | 9.7E+00 |
| Real target | $\cong$1.0E+07 | 1.8782E-03 | 0.0085 | 0.0001 | 9.4E+00 |
